# Visible-light-assisted degradation of crystal violet using CuO- and ZnO-incorporated (Am-*co*-BA)@PVA hydrogel nanocomposites

**DOI:** 10.1039/d6ra00342g

**Published:** 2026-03-10

**Authors:** Iltaf Uddin, Tanzil ur Rahman, Muhammad Said, Ezzat Khan, Muhammad Ishaq

**Affiliations:** a Department of Chemistry, University of Malakand Dir Lower Chakdara Khyber Pakhtunkhwa 18800 Pakistan; b National Centre of Excellence in Physical Chemistry, University of Peshawar Peshawar-25120 Pakistan; c Department of Chemistry, College of Science, University of Bahrain Main Campus Sakhir 32038 Kingdom of Bahrain ezkhan@uob.edu.bh; d Shaoxing Research Institute of Renewable Energy and Molecular Engineering, Shanghai Jiao Tong University Shaoxing 312000 China

## Abstract

Industrial effluents containing dyes such as crystal violet (CV) have adverse environmental effects due to their chemical inertness, toxicity and nonbiodegradability. Conventional separation techniques used to remove these pollutants are often inefficient; however, photocatalytic degradation using hydrogel photocatalysts is an effective and sustainable approach for wastewater treatment. CuO and ZnO nanoparticles (NPs) were successfully synthesized *via* a common co-precipitation method. The prepared metal oxide NPs were then incorporated into the hydrogel matrix to form hydrogel nanocomposites. For hydrogel preparation, polyvinyl alcohol (PVA) was used as a polymer, acrylic amide (Am) and butyl acrylate (BA) were used as monomers, and ammonium persulphate (APS) was used as an initiator. The successful fabrication of the hydrogel nanocomposite was verified using FTIR spectroscopy, XRD, SEM, and Brunauer–Emmett–Teller (BET) analysis. From FTIR spectroscopy data, the interaction and cross-linking of monomers and the polymer matrix were confirmed. The average crystallite size and uniform incorporation of metal oxide NPs into the hydrogel network were studied using XRD parameters. SEM images showed that after the integration of spherical-shaped metal oxide NPs into the hydrogel network, the surface of the hydrogel nanocomposite became rough and stratified, and the BET results indicated that the specific surface areas of ZnO- and CuO-doped hydrogel composites were 4.0835 cm^2^ g^−1^ and 4.9142 cm^2^ g^−1^, respectively. The photocatalytic activity of the synthesized hydrogel nanocomposites was investigated using an initial crystal violet (CV) concentration of 5 ppm to evaluate their degradation efficiency under visible light irradiation. The results showed that within an irradiation time of 110 min, the photocatalytic removal efficiency of CV reached 92.86% for the ZnO-doped hydrogel nanocomposite and 94.21% for the CuO-doped hydrogel nanocomposite at pH 9 using 0.01 g of the photocatalyst under visible light irradiation. The photocatalytic activity followed pseudo-first-order kinetics with rate constants of 0.0154 min^−1^ and 0.0148 min^−1^ for CuO- and ZnO-doped hydrogel nanocomposites, respectively. Furthermore, scavenging experiments showed that ˙OH radicals were the prominent species responsible for the degradation of CV. In this study, metal oxide-doped hydrogel nanocomposites were explored as sustainable and efficient photocatalysts for environmental remediation. The synthesized materials exhibited promising efficacy for the treatment of dye-contaminated wastewater.

## Abstract 2

Degradation of crystal violet dye was carried out using CuO-/ZnO-incorporated (Am-*co*-BA)@PVA hydrogel nanocomposites under visible light. The hydrogels exhibited enhanced surface area, efficient charge separation and superior photocatalytic activity.

## Graphical abstract

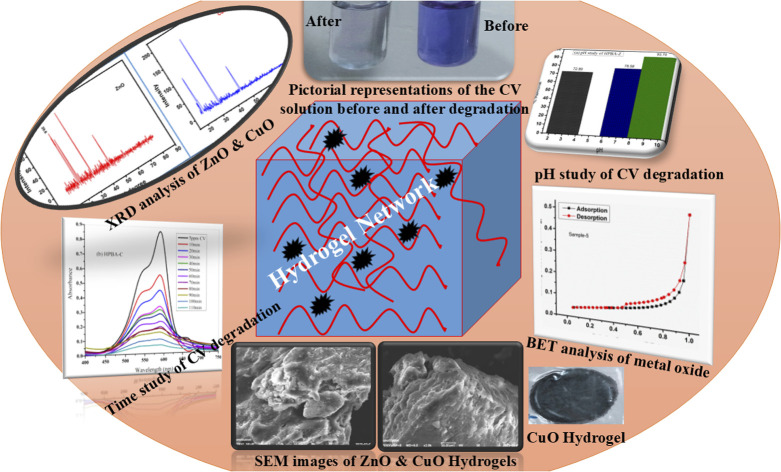

## 1 Introduction

Industrial and agricultural developments have made life easy, but the pollution caused by these developments is a serious threat. The effluents of these industries greatly deteriorate the ecological environment and human health. The most affected part of the globe is the hydrosphere, and different kinds of pollutants, especially dyes, are considered major contributors to its pollution.^1–3^

Dyes are organic compounds that impart color to the materials to which they are applied.^4^ These substances are mainly used in textile industries; hence, massive volumes of wastewater contaminated with these dyes are discharged during processing.^5^ These effluents have an adverse effect on the marine ecosystem even if they are present in very low concentrations.^6^ In addition, many of them, especially azo dyes, are considered carcinogenic. Azo dyes, after undergoing metabolic degradation, are reduced to aromatic amines, which are more harmful to the environment and human health.^7,8^ In humans, polluted water can cause water-borne diseases, such as cholera, typhoid, hepatitis A, diarrhea, vomiting and polio. Furthermore, when polluted water is consumed for a prolonged period, it leads to severe health problems, such as lung damage, kidney damage, neurotoxicity, cardiovascular issues, and even cancer.^9^

Among harmful dyes, CV is used for coloring leather, as an agent in Gram's staining, and in fertilizers, detergents, and bacteriostatic agents. The dye can cause severe problems, and it is considered a recalcitrant dye due to its nonbiodegradable nature;^10^ it can cause cancer in aquatic animals. CV also has a clastogenic nature; that is, it can break chromosomes, causing problems during the division process of affected cells.^11^

Thus, to lower the above-mentioned threats to the environment through wastewater, it is necessary to treat polluted water prior to its release into the aquatic environment. To fulfill the requirements for wastewater treatment, considerable efforts have been made, and separation techniques have been employed to remove effluents efficiently, which include developing advanced membranes,^12^ chemical oxidation,^13^ disinfection techniques,^14^ hybrid water supply systems,^15^ adsorption-based wastewater treatments using superabsorbents,^16^ and photocatalytic degradation.^2^ Among these techniques, photocatalysis is considered more efficient and effective due to its ecofriendliness, economic viability, and efficient degradation performance under visible light without producing secondary harmful waste materials. Developing photocatalysts with an optimal combination of light-absorption properties, electronic structures, and good separation of photo-generated charge carriers, such as TiO_2_, r-GO, ZnO, Cu_2_O, CdS, Fe_2_O_3_, and g-C_3_N_4_, is the core interest of current researchers.^17–21^

There are some demerits of using photocatalysts, like the agglomeration of the nanocatalyst, a low capacity for adsorption, poor recyclability, poor reusability, and low separation of nano-sized materials from water.^22,23^ Novel materials and techniques are urgently required to overcome these issues. It has been shown that the immobilization of photocatalysts onto suitable substrates is an effective method to stop their agglomeration, simplify the separation process of nano-sized photocatalysts, and provide an active medium for photoreactions to occur, making them suitable for large-scale applications.^24^ So far, different substrates, both organic and inorganic, have been employed as supports for the immobilization of photocatalysts, including glasses, films, fibers, carbon materials, polymers, ceramics, zeolites, and gels.^25–31^ The limitation of strains like high cost and weak adherence between photocatalyst and inorganic substrates have diverted the interest of researchers towards polymer supports.

Among polymer supports, hydrogels are considered promising substrate materials for photocatalysts. Hydrogels are macromolecules comprising water-swollen and cross-linked 3D polymeric networks synthesized by simple chemical reactions using one or more monomer/polymer/cross-linker units. These units, after gelation, form water-insoluble materials with a tendency to retain large amounts of water and biological fluids in the swollen state for a long time.^32^ The worldwide applications of hydrogels as substrates are due to their high porosity, biodegradability, and low cost. Furthermore, the different functional groups and cross-linking in the network of hydrogels provide stable sites for photocatalysts and allow dyes to concentrate into 3D networks, thereby increasing the efficiency of dye removal.^33–35^ Therefore, combining photocatalysts and hydrogels may be ideal to remove organic dyes from polluted water.

This article reports the development of two types of novel multifunctional hydrogel nanocomposites by the incorporation of CuO and ZnO nanoparticles within a flexible (Am-*co*-BA)@PVA interpenetrating polymer network, which provides synergistic charge transfer and increased visible light response. The Am-*co*-BA copolymer network enhances the swelling ability, hydrophilicity, and dispersion of pollutants, while the PVA backbone reinforces the mechanical property and provides stability under light illumination. The synthesized materials are characterized thoroughly by FTIR, XRD, SEM, and BET analyses to confirm their compositions, surface morphologies, and surface properties. The photodegradation performance of the photocatalysts is assessed against crystal violet (CV) dye under visible light illumination to evaluate their degradation efficiency, kinetics, and recyclability, giving insights into their potential for environmental remediation.

The integration of butyl acrylate (BA) into the acrylamide-PVA polymer system is tactically designed to introduce hydrophobic domains within the hydrogel network, which improve dye–matrix interactions and enhance photocatalytic efficiency by affording a higher degree of dye adsorption. The focus of the present study is the development of a multifunctional hydrogel nanocomposite with enhanced stability, stretchability, flexibility, and photocatalytic performance. Therefore, butyl acrylate contributes to the enhancement in the flexibility and mechanical robustness of the interpenetrating polymer network, which is a constructive approach for repeated photocatalytic application.

## 2 Experimental section

### 2.1 Materials

Zinc nitrate hexahydrate (Zn(NO_3_)_2_·6H_2_O, 99%), sodium dodecyl sulfate (SDS, 99.9%), ammonium carbonate trihydrate (NH_4_)_2_CO_3_·3H_2_O, absolute ethanol (C_2_H_5_OH, 99.9%), sodium hydroxide pellets (99.8%), sodium chloride (99.5%), and copper sulfate pentahydrate (CuSO_4_·5H_2_O, 99%), ammonium persulfate (APS, 98.5%), acrylamide (Am, 99.0%), polyvinyl alcohol (PVA, 90%), butyl acrylate (BA, 99%), crystal violet (CV, 99%), distilled water, and hydrogen peroxide (H_2_O_2,_ 98.9%) were used. All these chemicals were purchased from Sigma Aldrich in analytical grade and were used without further purification.

### 2.2 Synthesis of nanoparticles

CuO and ZnO nanoparticles were synthesized according to a previously described technique with minor modifications.^36^ CuO nanoparticles were synthesized *via* a hydrothermal process involving a heterogeneous reaction in an aqueous medium, carried out at the boiling temperature of water under high pressure exceeding 1 bar in a sealed autoclave. Initially, 2.814 g of copper sulfate was dissolved in 300 mL of distilled water to prepare a 0.0375 M solution. Then, 150 mL of a 0.15 M NaOH solution was added dropwise to the copper sulfate solution within 30 min with continuous stirring to prepare the Cu(OH)_2_ precipitate.

In the next phase, 200 mL of a mixed solution was poured into a 250 mL Teflon-lined stainless-steel autoclave, which was placed in an electric oven at 120 °C for 10 h. After allowing the mixture to cool gradually, a light blue solid was obtained. The blue solid was filtered and washed thoroughly with distilled water and ethanol several times to remove the unreacted materials. The wet gel of Cu(OH)_2_ was dried by heating in an oven at 80 °C for 24 h. The CuO nanoparticles were obtained by calcining Cu(OH)_2_ at 300 °C for 4 h in an electric furnace.

### 2.3 Synthesis of ZnO nanoparticles

ZnO nanoparticles were synthesized using Zn(NO_3_)_2_·6H_2_O and (NH_4_)_2_CO_3_·3H_2_O as precursors. A 0.0625 M Zn(NO_3_)_2_ solution was prepared by dispersing 3.7138 g of Zn(NO_3_)_2_·6H_2_O in 200 mL of distilled water. At the same time, a 0.0625 M (NH_4_)_2_CO_3_ solution was prepared by adding 2.248 g of (NH_4_)_2_CO_3_·3H_2_O in 240 mL of distilled water under ambient conditions and stirring for 15 min. The ammonium carbonate solution was added dropwise to the zinc nitrate solution with continuous stirring for 1 h. The combined crystals of zinc carbonate were finally vacuum-filtered and washed thoroughly with deionized water and ethanol to remove byproducts like NH_4_NO_3_, (NH_4_)_2_CO_3_ residues, and solvents. The collected crystals of ZnCO_3_ were kept overnight at 80 °C in an oven to remove excess particles and readily volatile materials including solvents. Finally, the dry ZnCO_3_ was calcined at 500 °C for 2 h.

### 2.4 Fabrication of the CuO-incorporated hydrogel composite

The fabrication of the CuO nanoparticle-incorporated hydrogel was initiated by the free radical polymerization mechanism using a homogeneous mixture of acrylamide (AA), butyl acrylate (BA) and polyvinyl alcohol (PVA) in distilled water. First, micelles were formed in 8 mL of DI water by adding 0.45 g of SDS and 0.20 g of NaCl under continuous stirring. Then, the hydrophobic monomer, BA (1 mL), was added to the solution, followed by the addition of 1.5 g of Am to the mixture. When the solution became clear, 5% PVA (0.075 g) was added, and the mixture was heated to 70 °C to dissolve PVA. Then, 0.01 g of CuO nanoparticles was dispersed in 2 mL of DI water in a sonicator for 2 h, which was added to the reaction mixture to get a homogeneous mixture. Finally, 0.05 g of ammonium persulphate (APS) was dissolved in 1 mL of DI water and added to the mixture as a free radical initiator and placed in an oven at 60 °C for 45 min for polymerization. The synthesized CuO hydrogel was then washed with DI water to remove the unreacted material and SDS, dried and stored for further use; the material was labeled as HPBA-C.

### 2.5 Fabrication of the ZnO-incorporated hydrogel composite

**Scheme 1:**
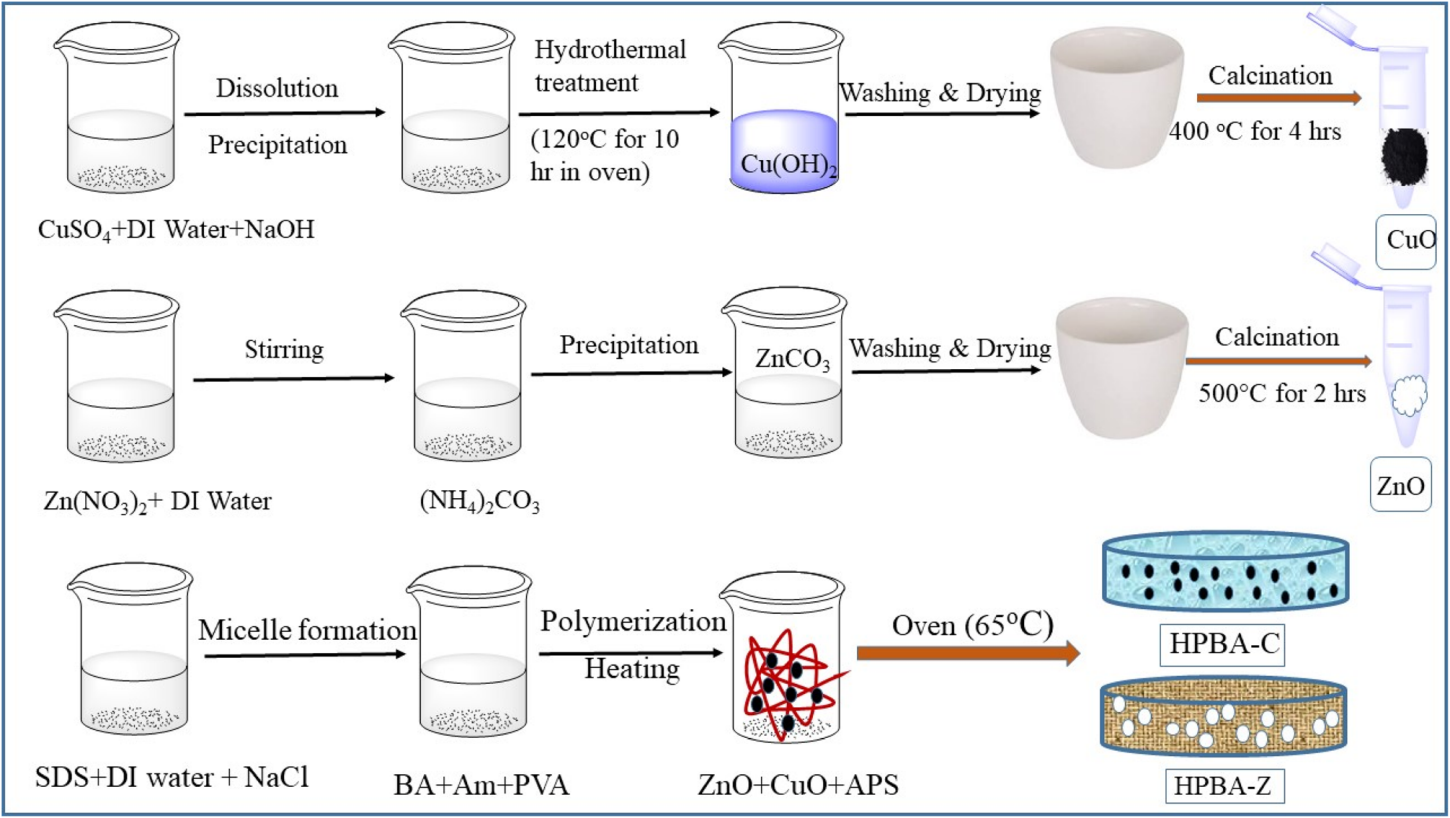
Schematic showing the co-precipitation methods for the preparation of CuO and ZnO NPs and the steps involved in their smooth incorporation in the hydrogel network to prepare hydrogel nanocomposites (HPBA-C and HPBA-Z, respectively).

**Fig. 1:**
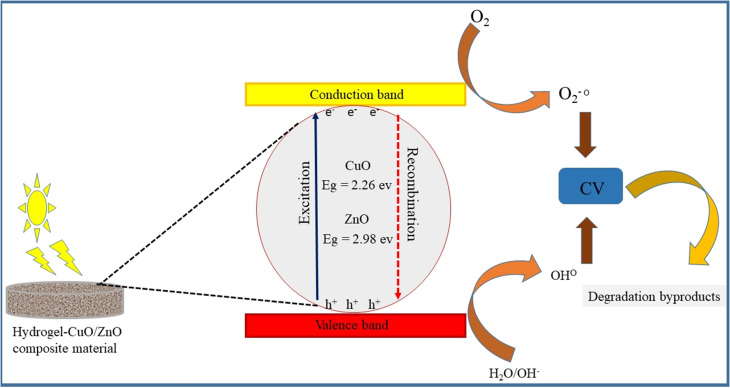
Graphical representation depicts the formation of the interpenetrating polymer network (IPN) (Am-*co*-BA)@PVA, the possible physical crosslinking among the polymer chains, and the incorporation of metal oxides (ZnO and CuO) into the polymer network.

The fabrication of the ZnO nanoparticle-incorporated hydrogel was initiated by the free radical polymerization mechanism using a homogeneous mixture of Am, BA and PVA in water. Micelles were formed in 8 mL of DI water by adding 0.45 g of SDS and 0.20 g of NaCl under continuous stirring. The hydrophobic monomer, BA (1 mL), was added to the solution, followed by the addition of 1.5 g of Am to the mixture. Under continuous stirring, after the solution became clear, 5% PVA (0.075 g) was added, and the mixture was heated to 70 °C to dissolve PVA. ZnO nanoparticles (0.01 g) were dispersed in 2 mL of DI water in a sonicator for 2 h and then added to the mixture to get a homogeneous solution. The free radical initiator, ammonium persulphate (0.05 g), was dissolved in 1 mL of DI water, added to the mixture and placed in an oven at 60 °C for 45 min for polymerization. The synthesized ZnO hydrogel was thoroughly washed with DI water to remove unreacted materials and SDS, dried and stored for further use, and it was labelled as HPBA-Z. The synthetic scheme of photocatalysts and metal oxide-based hydrogel composites is shown in Scheme 1. A graphical representation of the possible interactions inside the hydrogel network is shown in Fig. 1.

### 2.6 Characterization of metal oxide-incorporated hydrogel composites

The FTIR spectra of HPBA-C and HPBA-Z composites were analyzed by a Nicolet iS50 spectrometer. The crystalline phase structures of the materials were determined by powder X-ray diffraction (XRD) using a Bruker D8x instrument. The surface topography and internal section morphology of the composites were determined by SEM (6700, Bruker, Germany). The BET analysis was carried out to measure the surface area and porosity of the materials.

### 2.7 Photocatalytic degradation of dyes (crystal violet)

The dye CV, also known as Gentian violet and methyl violet, is a triaryl methane basic dye with the IUPAC name tris(4-(dimethylamino)phenyl)methylium chloride. CV has been widely used in different fields, such as pharmaceutical, textile and agricultural industries, for various purposes. It is known to be a stubborn chemical that has adverse effects on aquatic life.^37^ The photocatalytic degradation of CV was carried out under visible light. The photocatalyst (0.01 g) and 20 mL of a 5 ppm CV solution were mixed and stirred for 40 min in a dark environment to attain the dye adsorption equilibrium with the photocatalyst, and the mixture was then kept under visible light to start the photocatalytic dye degradation process. A dye sample was taken every 10 min and filtered through a microfilter, and the concentration of CV was determined by measuring the UV absorbance of the dye-containing solution. The degradation capacity and kinetics of the photocatalyst were determined under different pH conditions (3, 7, and 9) and at varying catalyst doses. The degradation capacity of the photocatalyst was measured using the following equation:

1 Degradation capacity (DC) = (*A*_0_ − *A*_i_)/*A*_0_ × 100

where *A*_0_ and *A*_i_ are the initial and equilibrium absorbance of the solution, respectively.

## 3. Results and discussion

### 3.1 Characterization of CuO- and ZnO-based hydrogel composites

#### 3.1.1 Fourier transform infrared (FTIR) spectroscopic analysis

**Fig. 2:**
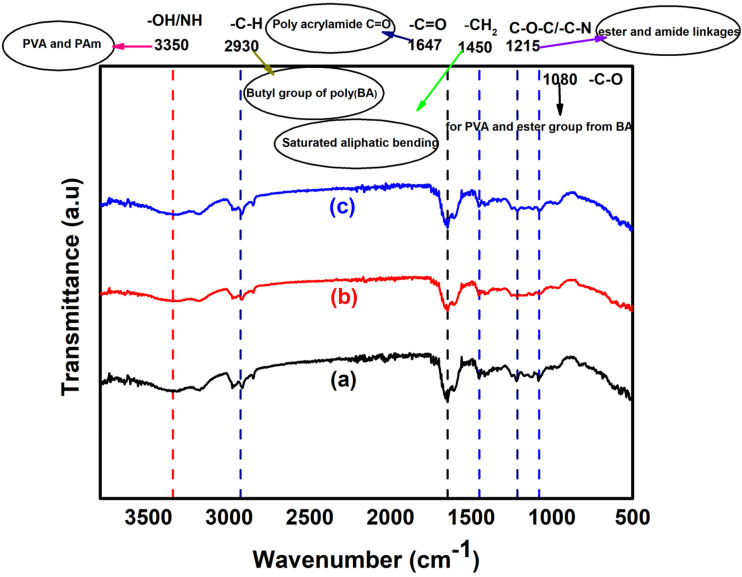
FTIR spectra showing the major absorption peaks in the (a) poly(Am-*co*-BA)@PVA (PBA), (b) CuO-incorporated hydrogel composite (HPBA-C) and (c) ZnO-incorporated hydrogel composite (HPBA-Z).

The FTIR spectra of the pure hydrogel, CuO-incorporated hydrogel composite and ZnO-incorporated hydrogel composite are shown in Fig. 2a, b and c, respectively. FTIR gives useful information regarding the formation of the final material. The shift, disappearance, broadening and appearance of new peaks are significant indicators used to check the formation of new bonds or different types of interactions among the components of the synthesized material. In the spectrum, the characteristic peaks in the range of 3150–3350 cm^−1^ are assigned to –OH and –NH stretching vibrations present in PVA and polyacrylamide. The prominent peak at 2930 cm^−1^ is attributed to aliphatic C–H stretching vibrations, whereas that appearing at 1450 cm^−1^ is assigned to –CH_2_ bending. Furthermore, the peak at 1647 cm^−1^ clearly indicates the presence of –C=O stretching, while the peaks at 1215 cm^−1^ and 1080 cm^−1^ are predominantly attributed to C–O–C or C–N and C–O stretching vibrations, respectively.^38^ There is distinct evidence in the FTIR spectra (Fig. 2a) that strongly supports the successful formation of the interpenetrating polymer network hydrogel using PVA, Am, and BA as reaction components. The disappearance of the =C–H group in the range of 1630–1640 cm^−1^ supports the polymerization of Am monomers. The broadening of the –OH/–NH stretching peak at 3350 cm^−1^ shows strong H-bonding, further supporting the physical interaction between PVA and PAm. The presence of a strong stretching peak at 1647 cm^−1^ confirms the presence of the acrylamide network within the material. Furthermore, the IR peaks at 1215 and 1080 cm^−1^ confirm the successful incorporation of poly(butyl acrylate) in the polymer network.

A slight shift of peaks in the O–H/N–H regions is observed when CuO and ZnO are incorporated into the polymer matrix, implying H-bonding or electrostatic interactions between metal oxides and the hydrogel network. The minor variations in the peak intensities at around 1647 and 1215 cm^−1^ (Fig. 2b and c) may show the weak interactions of the carbonyl/amino groups of the hydrogel with the metal oxide nanoparticles. Finally, the absence of any new peak in the spectra supports the physical encapsulation of metal oxide NPs in the hydrogel network.

The added butyl acrylate (BA) monomers build localized hydrophobic microdomains within the polymer network of hydrophilic groups. These segments support improved dye–matrix interactions *via* hydrophobic association, thus enhancing the adsorption of dye molecules prior to their photocatalytic degradation. This synergic effect between adsorption and photocatalysis enhances the overall photocatalytic efficiency for dye degradation of the presented system.

#### 3.1.2 Structural morphology

**Fig. 3:**
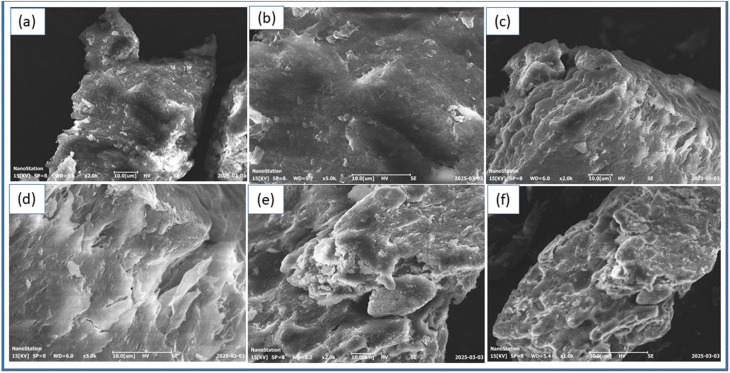
Cross-sectional SEM micrographs of the (a and b) pure hydrogel, (c and d) ZnO-doped hydrogel nanocomposite (HPBA-Z) and (e and f) CuO-doped hydrogel nanocomposite (HPBA-C). The cracked cross-sections show an uneven and stratified internal morphology with a uniform dispersion of metal oxide NPs within the hydrogel matrix. The magnification details and scale bars are shown in each image.

The morphological features of ZnO- and CuO-doped hydrogel materials were uncovered using SEM. This technique is an important characterization tool for examining the morphological properties of the design material, such as porosity, surface texture, and crystal grain appearance.^39^ The SEM micrographs of hydrogels without any NPs are shown in Fig. 3a and b, those of the ZnO-doped hydrogel are shown in Fig. 3c and d, and those of the CuO-doped hydrogel are shown in Fig. 3e and f. As seen in Fig. 3a and b, the surface of the hydrogel network appears to be smooth, less porous and compact. In the SEM images (Fig. 3c and d), the surface appears to be rough, porous, wrinkled, and compact, indicating the layer-by-layer integration of ZnO within the polymer matrix. These surface textures suggest that ZnO NPs are well-incorporated inside the polymer matrix, producing pores and microchannels that increase the interaction of light and dyes with the surface of the gel material. Furthermore, the stratified structure in Fig. 3c reveals the incorporation of ZnO in suitable amounts and the interfacial compatibility between ZnO and the polymer network. These properties further enhance the photocatalytic activity of the material by promoting the mobility of electrons and preventing charge recombination. In Fig. 3e and f, the plate-like structures show the dispersion of CuO nanoparticle clusters within the hydrogel networks. The distribution of pores and interstitial gaps of different sizes throughout the polymer network is an important factor for the effective adsorption of dye molecules within the polymer matrix, which further enhances the photocatalytic degradation efficiency. The obtained SEM micrographs showing fractured edges and a layered interior shape relate to the cross-sectional views of the hydrogel nanocomposites. The micrographs clearly show the layered and porous internal structure of the hydrogel network, which further confirms the uniform dispersion of metal oxide NPs within the hydrogel matrix.

#### 3.1.3 Brunauer–Emmett–Teller (BET) study

**Fig. 4:**
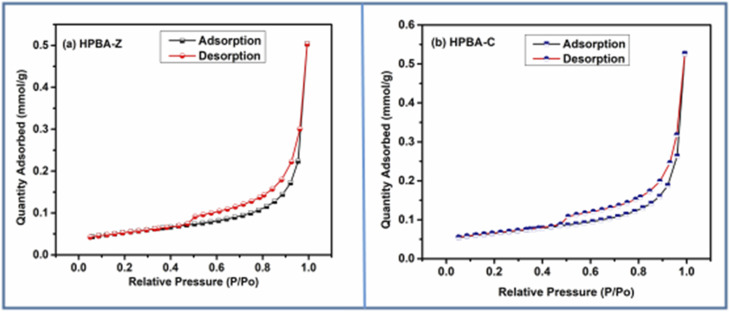
N_2_ adsorption and desorption isotherms of the (a) ZnO-based hydrogel nanocomposite (HPBA-Z) and (b) CuO-based nanocomposite (HPBA-C). Hysteresis loops in the adsorption–desorption isotherms of both samples show characteristics indicative of mesoporous materials.

To further investigate the surface characteristics, like surface area, surface porosity, pore volume and pore size, of the hydrogels, BET analysis was performed. In the BET analysis, the surface characteristics of the samples were analyzed by N_2_ adsorption. Static nitrogen physisorption was applied to obtain the nitrogen adsorption–desorption isotherms using a Micromeritics Tristar II Plus surface area and pore analyzer.^40^Fig. 4a and b show the N_2_ adsorption–desorption isotherms of HPBA-Z and HPBA-C, respectively, while the surface parameters obtained from nitrogen adsorption–desorption isotherms are summarized in Table 1. From the table, it is evident that the BET surface area of HPBA-C (4.9142 m^2^ g^−1^) is slightly larger than that of HPBA-Z (4.0835 m^2^ g^−1^). This reveals that the integration of CuO NPs into the hydrogel network leads to a greater degree of surface roughness or porosity compared to ZnO. Furthermore, the total pore volume of HPBA-C is found to be 0.018255 cm^3^ g^−1^, and that of HPBA-Z is 0.017441 cm^3^ g^−1^.

**Table 1:** Data collected *via* BET analysis show the surface areas, pore volumes, and pore sizes of the metal oxide-doped nanocomposites

Samples	BET surface area (m^2^ g^−1^)	Pore volume (cm^3^ g^−1^)	Pore size (Å)
HPBA-Z	4.0835	0.017441	169.070
HPBA-C^a^	4.9142	0.018255	148.595

a CuO-doped nanocomposites show a slightly larger surface area than the ZnO-doped nanocomposites.

The presence of hysteresis loops in the adsorption–desorption isotherms of both samples shows characteristics indicative of mesoporous materials. The difference in the adsorption–desorption behaviors also reflects the distinct interaction mechanisms of ZnO and CuO with the polymeric chains of the hydrogel. These distinctions are important for the applications of such materials in the field of photocatalysis.

#### 3.1.4 X-ray diffraction (XRD) analysis

**Fig. 5:**
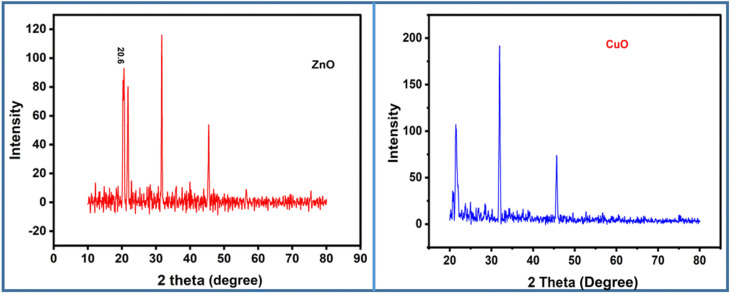
Clear and distinct powder XRD patterns show the successful incorporation of metal oxide (ZnO and CuO) NPs into the hydrogel network. The well-defined peaks further reveal the uniform distribution of NPs and the proper crystallite structures of metal oxides inside the hydrogel network. (Left) ZnO-doped hydrogel nanocomposite (HPBA-Z) and (Right) CuO-doped hydrogel nanocomposite (HPBA-C).

Powder XRD analysis was performed to investigate the crystalline phase of the synthesized hydrogel nanocomposites. Incorporation of ZnO NPs into hydrogel matrix are shown in Fig. 5a and CuO NPs embedded within the hydrogel network in Fig. 5b. As shown in Fig. 5a, the XRD pattern displays sharp and discrete diffraction peaks, indicating the crystalline nature of ZnO NPs within the hydrogel network. The most intense peak is observed at 2*θ* ≈ 36°, which resembles the (101) plane of hexagonal wurtzite of ZnO, consistent with JCPDS card no. 36-1451. Besides this sharp peak, additional peaks are also present at 2*θ* ≈ 31.8° and 34.4°, corresponding to the (100) and (002) planes, respectively. All these characteristic peaks confirm the incorporation of ZnO NPs and the retention of the crystalline nature of ZnO within the hydrogel network. Furthermore, the sharpness and intensity of the peaks imply a negligible interference from the amorphous nature of the hydrogel, suggesting the complete incorporation and structural integrity of the ZnO phase. The XRD pattern of CuO NPs in the hydrogel matrix is shown in Fig. 3b, which displays the characteristic peaks of monoclinic CuO, mostly prominent at 2*θ* ≈ 32.5°, 35.5°, and 38.7°, which correspond to the (110), (−111) and (111) planes, respectively (JCPDS card no. 48-1548). The clarity and intensity of the peaks in different positions indicate that CuO NPs remain crystalline within the polymer matrix. Compared to ZnO, the peaks of CuO are slightly broader, possibly due to its smaller crystallite size or partial amorphization during hydrogel incorporation. However, significant diffraction peaks remain distinct, confirming the structural integrity of the CuO crystalline phase in the hydrogel matrix. The XRD patterns of both NPs in hydrogel networks show that metal oxide NPs are successfully incorporated into the hydrogel network without substantial loss in their crystallinity. The distinct and well-defined diffraction peaks for both ZnO and CuO suggest their good dispersion, proper crystallite size, and potential interactions with the hydrogel network. These structural features highlight their suitability for applications in diverse fields, such as antimicrobial, photocatalytic, and sensing applications, where high structural stability and functional performance are required.

The crystallite sizes of the CuO- and ZnO-doped hydrogel nanocomposites were determined using the Scherrer equation (*D* = *Kλ/*(*β* cos *θ*)) based on the most intense diffraction peaks. The CuO- and ZnO-doped hydrogels exhibit crystallite sizes of 23.1 nm and 28.8 nm, respectively (Table 2). The achieved diffraction patterns match well with standard JCPDS files (CuO: 80-1916; ZnO: 36-1451), confirming their monoclinic and wurtzite phases of CuO and ZnO, respectively, and indicating high phase purity. The nanoscale crystallite dimensions imply the uniform incorporation of metal oxide nanoparticles within the hydrogel matrix, contributing to enhanced photocatalytic performance.

**Table 2:** XRD parameters and crystallite sizes of CuO- and ZnO-doped hydrogel nanocomposites calculated using the Scherrer equation, confirming their nanoscale crystallinity and phase purity

Sample	2*θ* (°)	FWHM (°)	*θ* (°)	*β* (rad)	*K*	*Λ* (nm)	*D* (nm)	Remarks
CuO–Hydrogel	31.9409	0.35899	15.9705	0.00627	0.9	0.15406	23.1	Monoclinic CuO phase
ZnO–Hydrogel	31.6900	0.28724	15.8450	0.00501	0.9	0.15406	28.8	Wurtzite ZnO phase

### 3.2 Photocatalytic degradation of CV

**Fig. 6:**
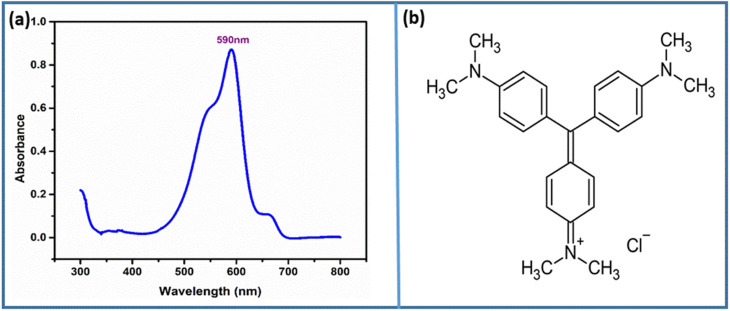
(a) UV-Vis absorption spectrum of CV showing the characteristic absorption maximum at 590 nm prior to photocatalyst addition and (b) molecular structure of CV.

To study the photocatalytic degradation of the CV dye using CuO and ZnO NP-dopped hydrogel nanocomposites under exposure to visible light, this study focused on the influence of several factors affecting the degradation process, including contact time, initial concentration of the dye, photocatalyst loading and pH of the medium. A 100 W xenon lamp was used as the light source for the photocatalytic degradation of the CV dye. The intensity of the incident light on the sample surface was measured to be approximately 1000 W m^−2^. The wavelength range of the xenon lamp was 350–800 nm, providing visible light conditions. The distance between the lamp and the surface of the photocatalyst suspension was maintained at 10 cm. The UV-vis spectrum of dye solution was determined at 590 nm before addition of photocatalyst aiming at easy comparison of possible change in absorption behaviour in afterward process (Fig. 6).

#### 3.2.1 Contact time study

**Fig. 7:**
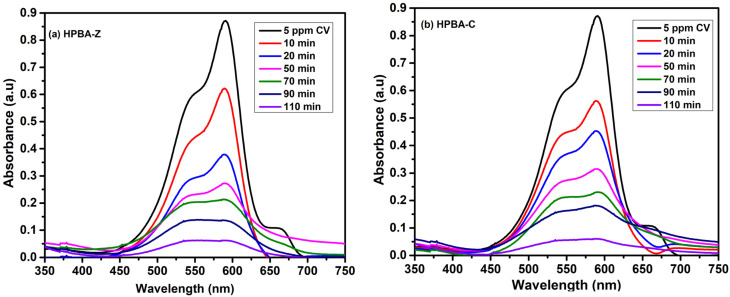
Effect of the irradiation time on the photocatalytic degradation of CV (dye concentration = 5 ppm, initial dye pH ≈ 7, and catalyst dose = 0.01 g) using the (a) ZnO-doped (Am-*co*-BA)@PVA hydrogel nanocomposite (HPBA-Z) and (b) CuO-doped (Am-*co*-BA)@PVA hydrogel nanocomposite under visible light illumination, showing a steady increase in the degradation efficiency with time and maximum removal after 110 min.

**Fig. 8:**
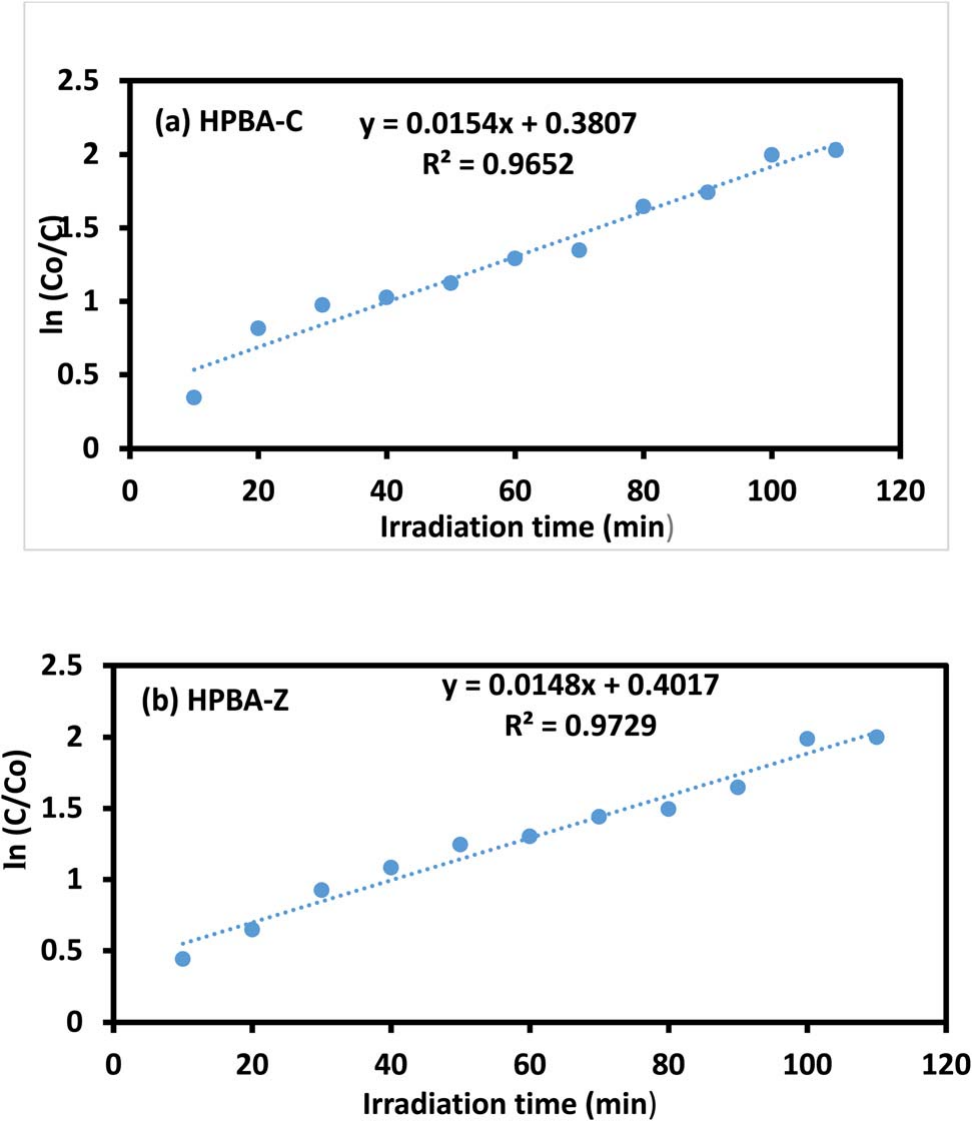
Apparent pseudo-first-order rate constants (*k*) calculated from the linear fits of ln(*C*_0_/*C*_*t*_) *vs.* time plots for the (a) CuO-doped (Am-*co*-BA)@PVA hydrogel nanocomposite (HPBA-C) and (b) ZnO-doped (Am-*co*-BA)@PVA hydrogel nanocomposite (HPBA-Z). HPBA-C exhibited a slightly higher k value than HPBA-Z, which is attributed to its narrower band gap and improved visible light absorption.

The adsorption of CV on the surface of the nanocatalysts, CuO-doped hydrogel nanocomposite (HPBA-C) and ZnO-doped hydrogel nanocomposite (HPBA-Z), was studied as a function of the contact time, which corresponds to the time of adsorption or the point of saturation of the adsorbent (photocatalyst) surface by the adsorbate (dye). The effect of the contact time on the degradation of a 5 ppm aqueous solution of CV using 0.01 g of HPBA-Z and HPBA-C is shown in Fig. 7a and b. The results reveal that the catalytic photodegradation of CV occurs. It is observed (Fig. 7a and b) that the photocatalytic activity of HPBA-C is slightly higher than that of HPBA-Z, primarily due to its lower band gap value. After an optimized irradiation time (110 min), HPBA-C shows 94.21% removal efficiency, while HPBA-Z shows 92.86% removal efficiency (Table 3). The degradation of the dye occurs at a higher rate in the initial stages due to the presence of more surface active sites on the catalyst. As photocatalysis proceeds, dye molecules or particles saturate the available sites on the catalyst surface, limiting the effective light exposure of the catalyst surface. The hydrogel matrix inherently has a high adsorption affinity toward dyes due to its porous structure, the presence of different moieties on its surface, and its large surface area. As a result, it effectively captures and concentrates molecules of CV on its surface, increasing the contact time between the dye and the catalyst. The increasing trend in the efficiency of dye removal with an increase in the exposure time is ascribed to the existence of more active sites on the catalyst surface, which helps the degradation reactions to occur,^41^ leading to greater removal efficiency. The rate constants of CuO-doped (HPBA-C) and ZnO-doped hydrogel composites (HPBA-Z) are shown in Fig. 8a and b, respectively. The recent advances in the field of photocatalysis of different organic dyes have revealed significant progress in the development of metal oxide-doped hydrogel photocatalysts. For example, the ZnO–polyacrylamide hydrogel photocatalyst shows a significant photocatalytic efficiency against MB, with more than 90% removal. Similarly, TiO_2_-gum acacia and CuO-PVA-based hydrogel nanocomposites have also shown pseudo-first-order rate constants in the range of 0.04–0.14 min^−1^, highlighting the role of the hydrogel network in enhancing the stability, surface reactivity, and consistent reusability of photocatalysts. A comparative analysis of recently developed metal oxide hydrogel photocatalysts and the nanocomposites presented in this study is provided in Table 4.

**Table 3:** Comparison of the photolysis, adsorption, and photocatalytic degradation of CV using ZnO- and CuO-based hydrogel nanocomposite (CV conc. = 5 ppm, initial dye pH ≈ 7, visible light source, irradiation time = 110 min, and catalyst dose = 0.01 g)

Parameters	Photolysis (light only)	ZnO hydrogel	CuO hydrogel
Catalyst dose (g)	—	0.01	0.01
Dye (CV) conc. (ppm)	05	05	05
Light source	100 W Xenon (visible)	100 W Xenon (visible)	100 W Xenon (visible)
Removal (%) – adsorption (dark)	—	∼6	∼6
Removal (%) – photolysis (light only)	<5	—	—
Removal (%) – photocatalysis	—	92.86	94.21
Rate constant *k* (min^−1^)	—	0.0148	0.0154

**Table 4:** Comparative analysis of the apparent rate constants (*k*) for dye degradation reported for different photocatalytic systems under different light sources and experimental conditions, together with the performance of the CuO- and ZnO-incorporated (Am-*co*-BA)@PVA hydrogel nanocomposites synthesized in the present study

S. no.	Photocatalyst	Dye	Apparent rate constant *k* (min^−1^)	Dye concentration (ppm)	Energy source	Ref.
1	Ag/g-C_3_N_4_	Methyl orange	0.0294	15	Visible	47
2	rGO@ZnO	Methylene blue	5.03 × 10^−3^	15	Sun light	48
3	TiO_2_–gum-acacia hydrogel	Rhodamine B	0.1356	10	UV lamp	49
4	TiO_2_–gum-acacia hydrogel	Methyl orange	0.1051	10	UV lamp	49
5	Ag_3_PO_4_/Ag/g-C_3_N_4_ heterojunction	Methyl orange	0.0413	10	Visible light, 300 W Xenon lamp	41
6	Zinc thin film	Reactive blue 19	0.006.9	10	UV lamp	50
7	ZnO	Reactive blue 19	0.0110	10	UV lamp	51
8	CuO-doped (Am-*co*-BA)@PVA hydrogel	Crystal violet	0.0154	5	Visible, Xenon lamp	***This study***
9	ZnO-doped (Am-*co*-BA)@PVA hydrogel	Crystal violet	0.0148	5	Visible, Xenon lamp	***This study***

#### 3.2.2 pH study

**Fig. 9:**
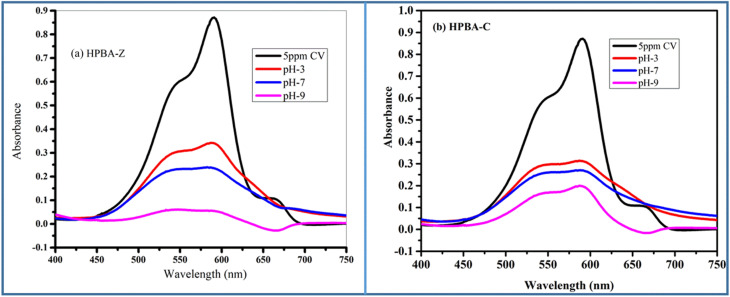
Effect of the pH on the photocatalytic degradation efficiency of crystal violet (CV) using the (a) ZnO-doped hydrogel nanocomposite (HPBA-Z) and (b) CuO-doped hydrogel nanocomposite (HPBA-C) under visible light irradiation (100 W Xenon lamp, dye conc. = 5 ppm, catalyst dose = 0.01 g, irradiation time = 110 min, and initial dye pH ≈ 7). The degradation efficiency increases with pH, reaching the maximum removal at pH 9 due to the enhanced generation of hydroxyl radicals and improved electrostatic attraction between dye molecules and the catalyst surface.

**Fig. 10:**
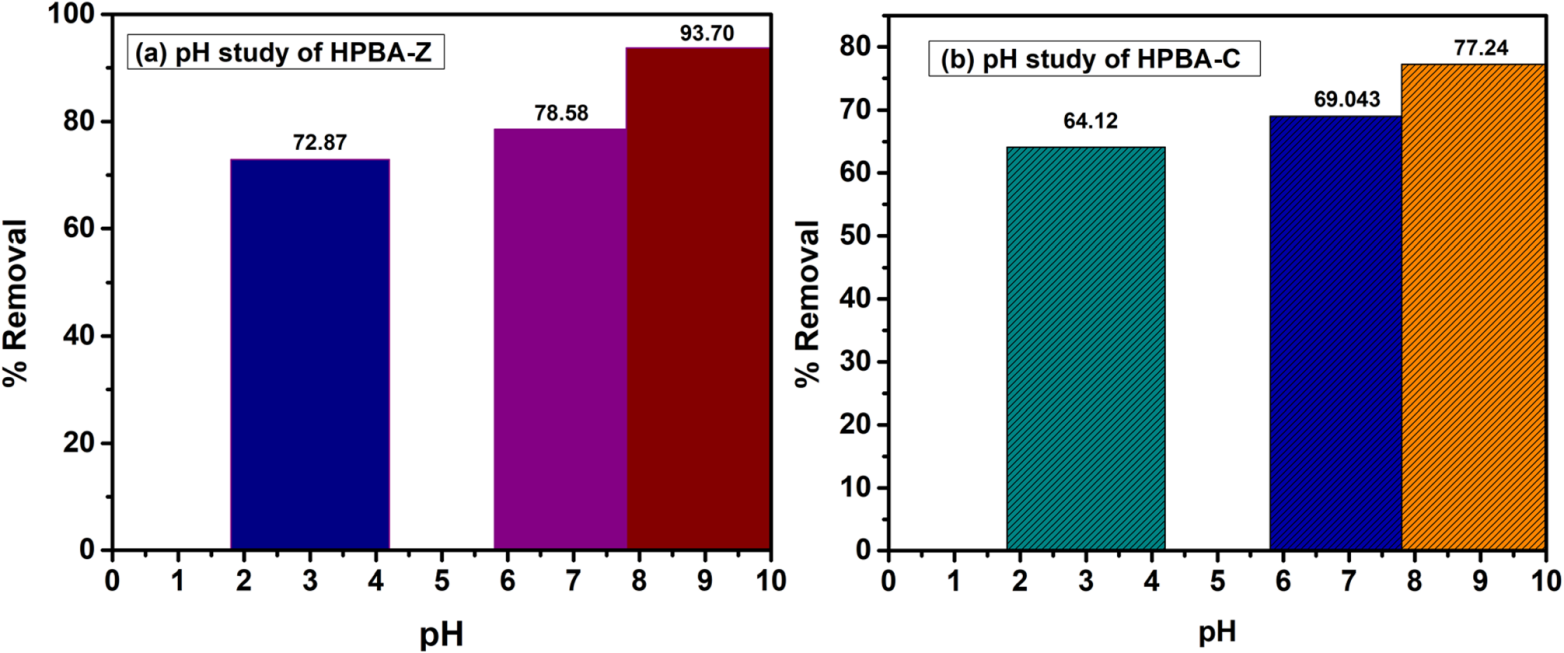
Bar graphs showing the effect of pH on the photocatalytic degradation of CV (dye conc. = 5 ppm, initial dye pH ≈ 7, visible light source, catalyst dose = 0.01 g, and irradiation time = 110 min) using the (a) ZnO-doped hydrogel nanocomposite (HPBA-Z) and (b) CuO-doped hydrogel nanocomposite (HPBA-C). Both photocatalysts show maximum degradation at pH 9.

The pH of a solution is another critical parameter in dye degradation reactions, as it hinders the adsorption efficiency of photoactive species.^42^ The pH of the solution containing the dye was adjusted to 3, 7 and 9 by adding HCl or NaOH (1 M) at a constant dye concentration of 5 ppm and a constant catalyst amount of 0.01 g. The percent removal of CV in solutions of various pH (3, 7 and 9) using HPBA-Z and HPBA-C photocatalysts is depicted in Fig. 9a, b and 10a, b. The study shows that the efficiency of photodegradation of the dye increases as the pH of the solution increases from 3 to 9. This is because in a basic medium, the surface of the photocatalyst becomes more negatively charged due an increased concentration of hydroxyl ions (OH^−^), which adsorb onto the surface and generate a negative surface potential. As a result, a strong electrostatic interaction is established between the negatively charged photocatalyst and the cationic dye. This electrostatic interaction further enhances the efficiency of adsorption of CV onto the photocatalyst, thereby increasing its degradation. Moreover, basic conditions favor the formation of hydroxyl radicals (˙OH), which are highly reactive and play a critical role in photocatalytic degradation processes. The low efficiency of the degradation process under acidic conditions can be ascribed to the electrostatic repulsion between the catalyst and cationic dye (CV); in addition to this effect, at lower pH, due to protonation (H^+^), the formation of hydroxyl ions decreases. The photocatalytic degradation efficiency of HPBA-C is 87.09%, 73.33% and 64.12% and that of HPBA-Z is 93.7%, 78.58% and 72.89% at pH 9, 7 and 3, respectively.

#### 3.2.3 Catalyst dosage study

**Fig. 11:**
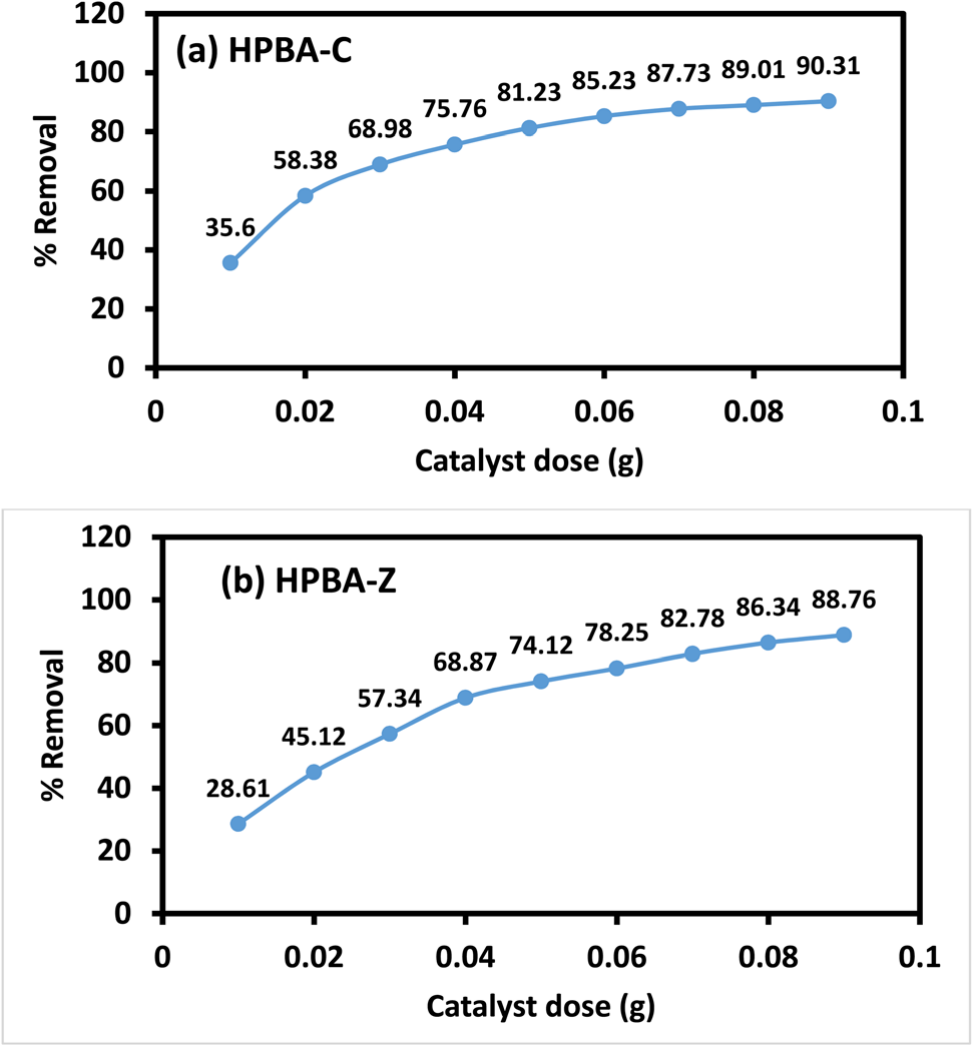
Effect of the catalyst dose on the photocatalytic degradation efficiency of CV (dye conc. = 5 ppm, initial pH ≈ 7, and visible light source) using (a) HPBA-C and (b) HPBA-Z, showing percent degradation for catalyst loadings of 0.01–0.09 g.

The photodegradation efficiencies stated in this section were obtained from the catalyst dosage study performed under fixed irradiation-time and experimental conditions to assess the effect of the catalyst loading. After the optimal-pH study, the effect of the catalyst dose on the degradation efficiency of CV was analyzed. As depicted in Fig. 11a and b, the dose of the catalyst was varied from 0.01 g to 0.09 g. The results show that as the dosage of the catalyst increases from 0.01 g to 0.09 g, the percent degradation also increases from 35.6% to 90.32% and 28.61% to 88.76% for HPBA-C and HPBA-Z, respectively.

This is because increasing the concentration of the catalyst enhances the number of active sites and surface area of the photocatalyst for dye molecules to adsorb onto. This further boosts the production of additional reactive species for dye degradation.^43^ It is clear from the Fig. 11a and b that the photodegradation increases in a dose dependent manner reaching to its highest value at the catalyst dose of 0.09 g for both HPBA-C and HPBA-Z. As the dosage increases, the photodegradation efficiency increases reaching to its optimum value of 0.09 g. This can be explained by the fact that at a higher catalyst loading, the efficacy of degradation decreases, probably due to the deactivation of active molecules by collision with the ground-state catalyst, thus reducing the rate of the photocatalytic reaction.^44^

#### 3.2.4 Initial dye concentration study

**Fig. 12:**
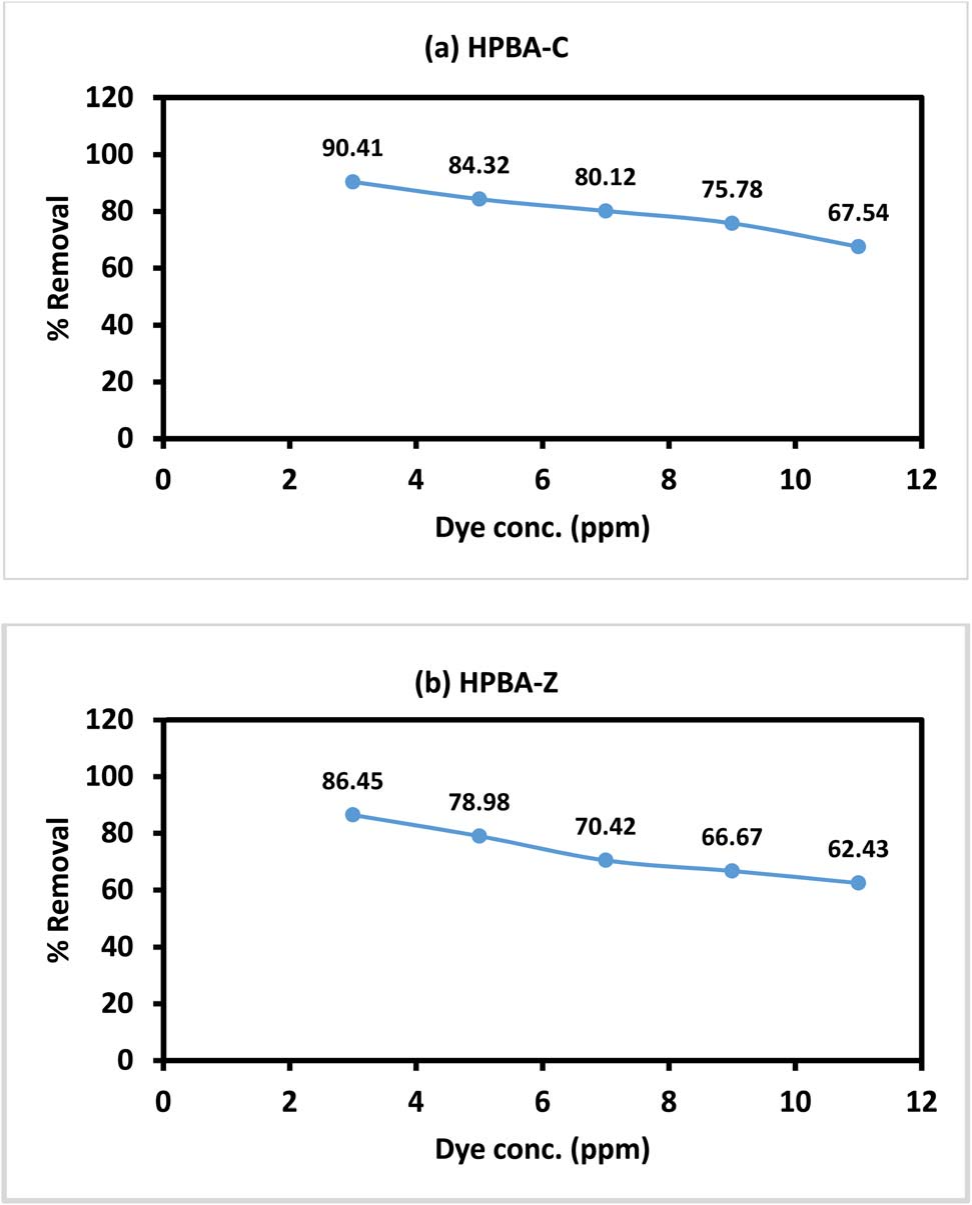
Effect of the initial dye concentration on the photocatalytic degradation performance of the (a) CuO-doped hydrogel nanocomposites (HPBA-C) and (b) ZnO-doped hydrogel nanocomposites (HPBA-Z) against CV (CV conc. = 5 ppm, initial pH ≈ 7, visible light source, and irradiation time = 110 min), showing a decreased removal efficiency at higher dye concentrations due to light absorption and active site saturation. Each experiment was conducted once under identical optimized conditions; reproducibility was confirmed through consistent instrumental measurements.

The activity of the photocatalysts in the degradation of CV with different concentrations (ranging from 3 to 11 ppm) was also evaluated. As shown in Fig. 12a and b, the catalytic degradation rate decreased with an increase in the concentration of the dye. At lower dye concentrations (3 and 5 ppm), the efficiency of photocatalytic degradation surpassed 85% for both catalysts, while at higher concentrations (>5 ppm), the degradation efficiency dropped to 70%. This was attributed to the limited number of active sites available for the degradation of an excess amount of the dye molecules. Furthermore, the high concentration of dye hindered the pathway of incoming photons, stopping them from reaching the surface of the photocatalyst and resulting in poor degradation efficiency.

#### 3.2.5 Reusability and stability study

**Fig. 13:**
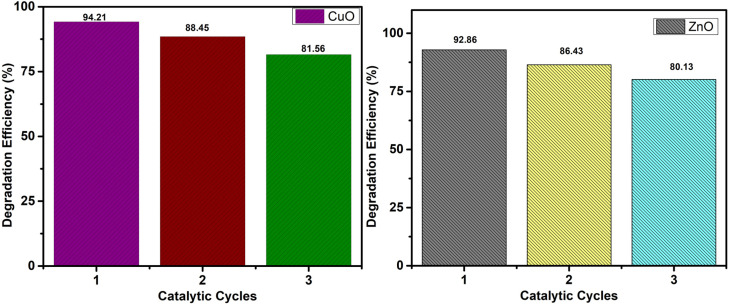
Recyclability and stability performance of the CuO- and ZnO-doped (Am-*co*-BA)@PVA hydrogel nanocomposites for the photocatalytic degradation of the selected dye (dye conc. = 5 ppm and initial pH ≈ 7) under visible light over three consecutive cycles. Both catalysts retained over 86% of their initial degradation efficiency after the third cycle, indicating their excellent stability and negligible loss in catalytic activity. Reproducibility was confirmed through consistent instrumental measurements.

The synthesized photocatalysts were tested to evaluate their recyclability and stability through repeated photocatalytic degradation cycles of CV under visible light irradiation. The photocatalysts were separated from the solution after each cycle, washed thoroughly with water and ethanol to remove any adsorbed dye molecules, then dried in oven at 60 °C, and used again in the next cycle. The experiment was performed for three consecutive cycles under the same experimental conditions of (pH 9, 0.01 g catalyst dose, 5 ppm dye concentration, and 100 W xenon lamp). The results (Fig. 13) showed only a minor decrease in the degradation efficiency after each cycle, indicating good recyclability and stability.

#### 3.2.6 Photocatalytic reaction mechanism

**Fig. 14:**
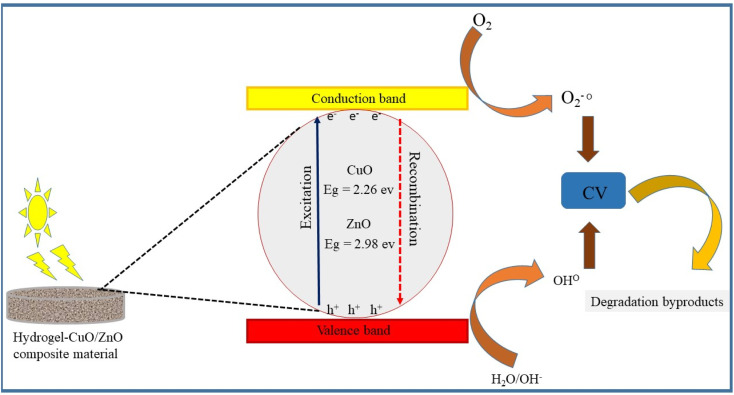
Photocatalytic reaction mechanism of the CuO-based (HPBA-C) and ZnO-based (HPBA-Z) hydrogel nanocomposites. Experimental conditions: CV conc. = 5 ppm, catalyst dose = 0.01 g, initial pH ≈ 7, and visible-light irradiation time = 110 min.

**Fig. 15:**
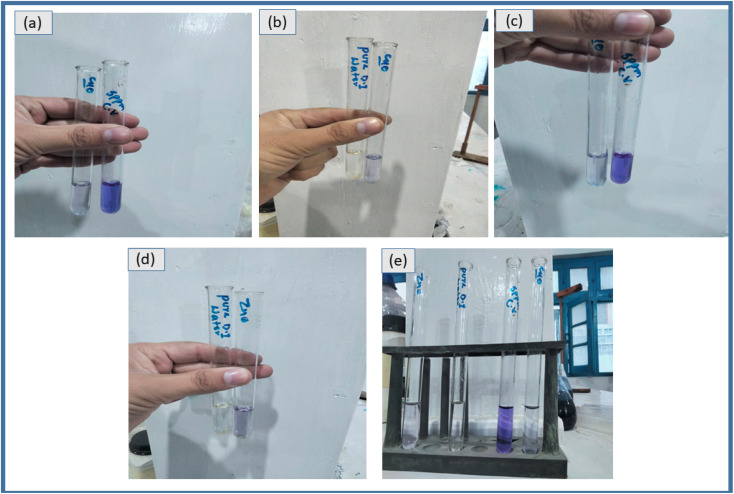
Photographs showing the photocatalytic degradation of CV under visible light irradiation using metal oxide-doped hydrogel nanocomposites. (a) CV solution before and after 110 min of irradiation in the presence of the CuO-doped hydrogel nanocomposite (HPBA-C). (b) Comparison of pure water and a CV solution after 110 min of irradiation in the presence of the CuO-doped hydrogel nanocomposite. (c) CV solution before and after 110 min of irradiation in the presence of the ZnO-doped hydrogel nanocomposite (HPBA-Z). (d) Comparison of pure water and a CV solution after 110 min of irradiation in the presence of the ZnO-doped hydrogel nanocomposite. (e) Comparative photocatalytic degradation efficiency of ZnO- and CuO-doped hydrogel nanocomposites toward CV. Experimental conditions: CV conc. = 5 ppm, catalyst dose = 0.01 g, initial pH ≈ 7, and visible-light irradiation time = 110 min.

During the photocatalytic degradation of dyes, hydroxyl radicals (˙OH) and photo-generated holes (h^+^) are recognized as the predominant reactive species, whereas superoxide radical anions (O_2_˙^−^) contribute only to a minor extent. The possible photocatalytic degradation mechanism of CuO- and ZnO-doped hydrogel composites is shown in Fig. 14. When the photocatalyst absorbs light, electrons are excited from the conduction band (CB) to the valence band (VB) thus leaving the same number of holes (h^+^) in the VB. Redox reactions on the catalyst surface are initiated by the separated holes and electrons, leading to the production of reactive oxygen species (ROS).^45,46^ The electrons in the CB interact with dissolved molecular oxygen and form superoxide anions, OH and so forth, while the positive holes combine with water molecules, leading to the formation of hydroxyl radicals (powerful oxidizing agents), see Fig. 15. All these ROS, predominently produced by CuO/ZnO within the hydrogel network, cause to degrade the dye into less harmful byproducts. The equations below show the overall mechanism of photocatalytic degradation reactions and apparent color change is shown in Fig. 15:CuO/ZnO HPBA + *hv* (visible radiation) → CuO/ZnO HPBA (e^−^ + h^+^)e^−^ + O_2_ → ˙O^−^h^+^ + H_2_O → H^+^ + ˙OHCV + (h^+^/˙O_2_^−^/˙OH) → harmless byproducts

#### 3.2.7 Apparent yield calculation

The apparent quantum yield (AQY) was calculated using the following relation:

2 AQY (%) = *N*_reacted molecules_/*N*_incident photons_ × 100

where *N*_reacted molecules_ was obtained from the moles of the dye degraded after photocatalysis and *N*_incident photons_ was obtained from the lamp intensity and irradiation time. The parameters required for this calculation are listed in Table 5.

**Table 5:** Parameters required for the calculations of AQY values for CuO- and ZnO-doped hydrogel nanocomposites

Parameters	Symbol	Values
Lamp power	*P*	100 W
Average wavelength	*λ*	500 nm
Irradiation time	*t*	110 min
Solution volume	*V*	20 mL
Initial dye concentration	*C*_0_	5 ppm
Molecular weight of CV	*M*	407.98 g mol^−1^
Degradation efficiency of CuO		94.21%
Degradation efficiency of ZnO		92.86%

The apparent quantum yield (AQY) was calculated using eqn (2) based on the number of dye molecules degraded and the total number of incident photons under visible light irradiation (*λ* = 500 nm and 100 W Xe lamp). The AQY values of CuO- and ZnO-doped hydrogel nanocomposites were found to be 0.0000084% and 0.0000083%, respectively.
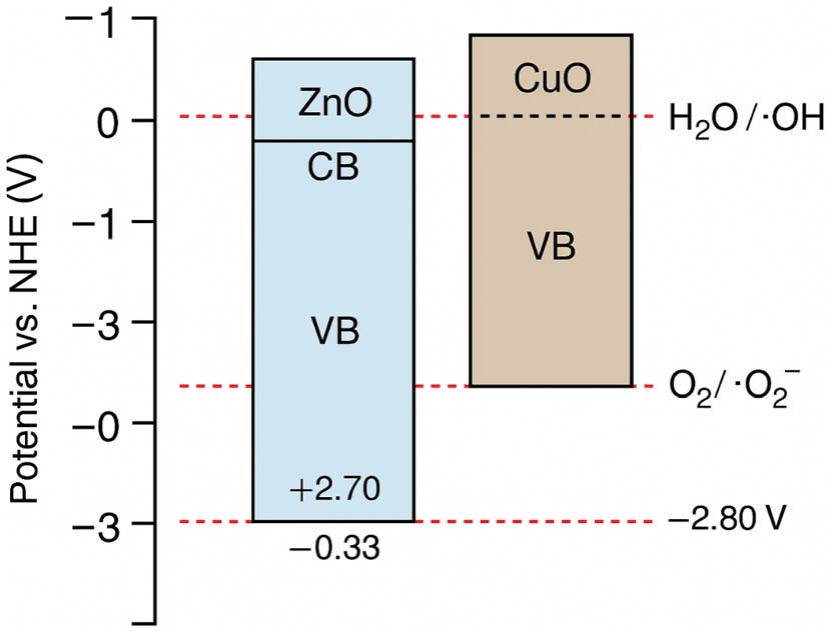


Literature-reported band-edge values were used to construct the energy band diagram and to interpret the possible charge transfer pathways and functional roles of ZnO and CuO within the heterostructured system.

## 4. Conclusion

The ZnO- and CuO-based hydrogel nanocomposites were synthesized and tested for their catalytic efficiency to degrade crystal violet dye under UV-vis light. The characterization techniques applied to analyze the materials were FTIR, XRD, SEM and BET analyses. The functionalities and cross-linking patterns among the polymer chains were studied through FTIR spectroscopy. The crystallite size and integration of NPs onto the polymer matrix were determined with the help of XRD. The SEM images showed that the hydrogel network became rougher upon NP incorporation. The pore size, pore volume and surface area were determined through BET analysis. Under visible light irradiation, HPBA-Z showed 92.86% degradation within a 110 min exposure time, while HPBA-C showed 94.21% degradation within the same time. It was observed through scavenging studies that among ROS, ˙OH radicals were the prominent species responsible for the degradation of the dye, followed by holes generated during the reaction. All results provide a practicable approach for ecofriendly and sustainable wastewater treatment applications.

Though the present research focuses on the effective visible-light-assisted photodegradation of CV using CuO- and ZnO-doped (Am-*co*-BA)@PVA hydrogel nanocomposites, various aspects permit further exploration. A comprehensive scavenger study to clearly identify the main reactive species involved in the photocatalytic mechanism was not performed in the present study and will be addressed in future work. Moreover, an efficient comparison with a single-monomer-based hydrogel network could offer deeper insights into the definite contribution of butyl acrylate to the adsorption–photocatalytic synergy.

## Author contributions

Experimental work, data interpretation, manuscript writing: IU; data collection and interpretation, manuscript writing: IU, TR and MI; and funds managements, conceptualization, data validation, supervision, manuscript writing, final draft reviewing: EK and MS.

## Conflicts of interest

The authors declare no conflicts of interest.

## Supplementary Material

RA-016-D6RA00342G-s001

RA-016-D6RA00342G-s002

## Data Availability

All data referenced and discussed in this manuscript are available from publicly accessible sources, including peer-reviewed publications and scientific databases, which are properly cited within the text. No new datasets were generated or analyzed during the current study. Supplementary information (SI) is available. See DOI: https://doi.org/10.1039/d6ra00342g.
