# Supplementary material for: Visible-light-assisted degradation of crystal violet using CuO- and ZnO-incorporated (Am-co-BA)@PVA hydrogel nanocomposites
Source: RSC Adv. 2026 Mar 10;16(15):13332–46. doi: 10.1039/d6ra00342g (PMC12973283; doi:10.1039/d6ra00342g)
Supplement: RA-016-D6RA00342G-s001 [file RA-016-D6RA00342G-s001.pdf]

This journal is © The Royal Society of Chemistry 2026

Supporting Information file for manuscript

Table S1. This table shows the major absorption peaks of FTIR for poly(Am-co-BA)@PVA, ZnO and CuO doped nanocomposite hydrogel network.

Wavenumber (cm^{-1})	Vibrations	Origin of peaks	Remarks
3350–3330	O–H and N–H stretching	PVA and polyacrylamide hydroxyl/amino groups	Broad band, indicates strong hydrogen bonding and cross-linked hydrogel network
2930–2915	C–H stretching (–CH_2–, –CH_3)	Butyl acrylate and PVA backbone	Confirms alkyl presence in polymer chain
1650–1647	C=O stretching (amide I)	Acrylamide units (polyacrylamide network)	Confirms amide incorporation; overlaps with ester C=O of BA
1450–1467	CH_2 bending / scissoring	Aliphatic chain deformation	Suggests flexible alkyl groups in hydrogel
1215–1218	C–O–C / C–N stretching	Ester and amide linkages	Indicates interpenetration and copolymer formation
1080–1081	C–O stretching	PVA and butyl acrylate ester groups	Confirms successful crosslinking of PVA chains
828–827	C–H out-of-plane bending	Alkene or substituted CH vibrations	Minor feature, confirms polymeric backbone integrity
580–590	Metal–O vibration	Cu–O or Zn–O stretching in HPBA–C and HPBA–Z	Confirms nanoparticle incorporation in hydrogel matrix

The relative shifts and intensity variations have been analyzed in detail. Specially, the broad –OH/NH stretching band at ~3350 cm^{-1} (from PVA and polyacrylamide) became slightly broadened and shifted to lower wavenumbers in the CuO- and ZnO-doped hydrogels, signifying hydrogen bonding interactions between the polymer matrix and the metal oxide nanoparticles. The C=O stretching vibration of the amide group (~1647 cm^{-1}) also shifted slightly to 1635–1638 cm^{-1}, representing coordination between the carbonyl oxygen and surface metal ions. The C–O–C and C–N stretching peaks at 1215–1080 cm^{-1} exhibited reduced intensity, further confirming successful crosslinking and metal oxide integration within the IPN network. These spectral changes collectively confirm that the integration of CuO and ZnO nanoparticles led to strong interfacial interactions with the polymer chains, supporting the formation of a stable and homogeneous hydrogel nanocomposite structure.

S2. Time study graphs of ZnO-doped and CuO-doped hydrogel nanocomposites, for degradation of 5ppm CV at 110 mints time irradiation under Xenon lamp.

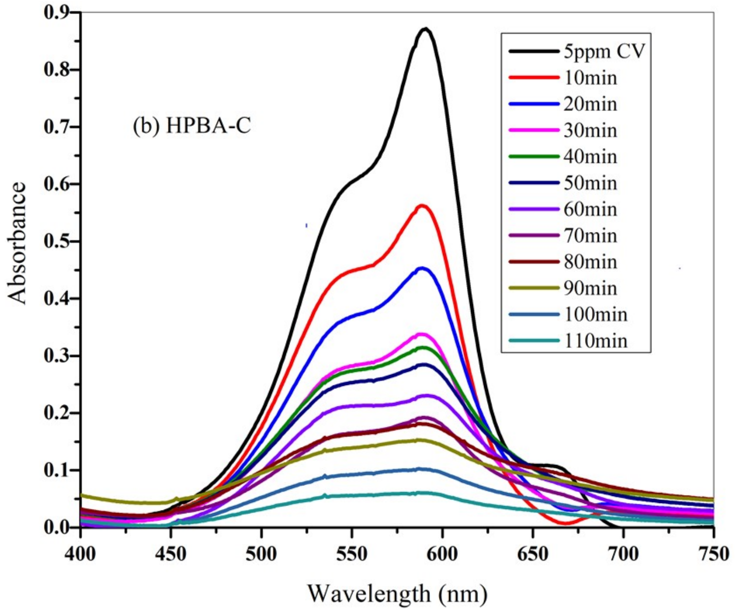

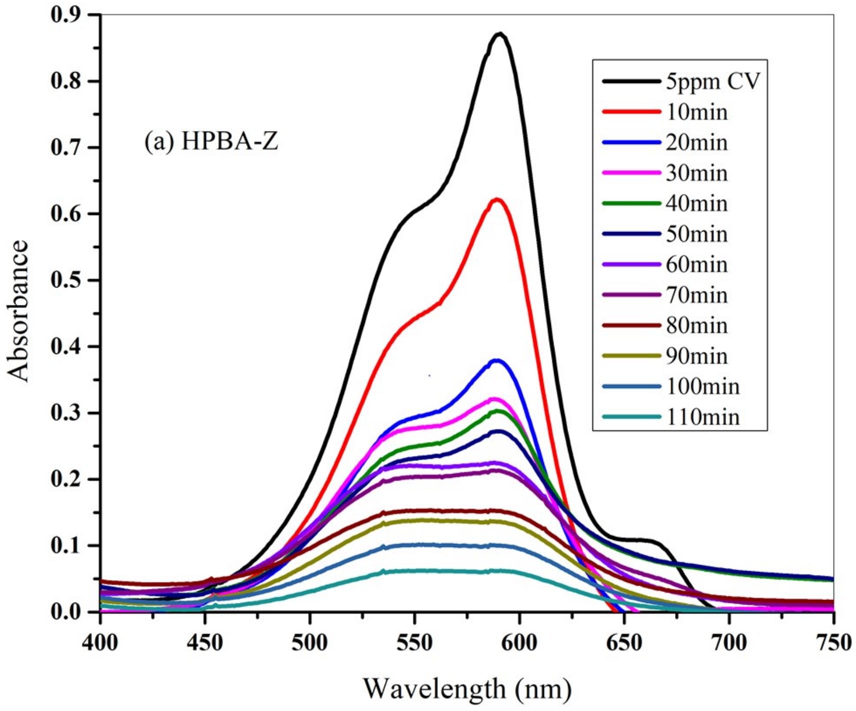

S3. SEM images of pure hydrogel, CuO-doped hydrogel and ZnO-doped hydrogel nanocomposites.

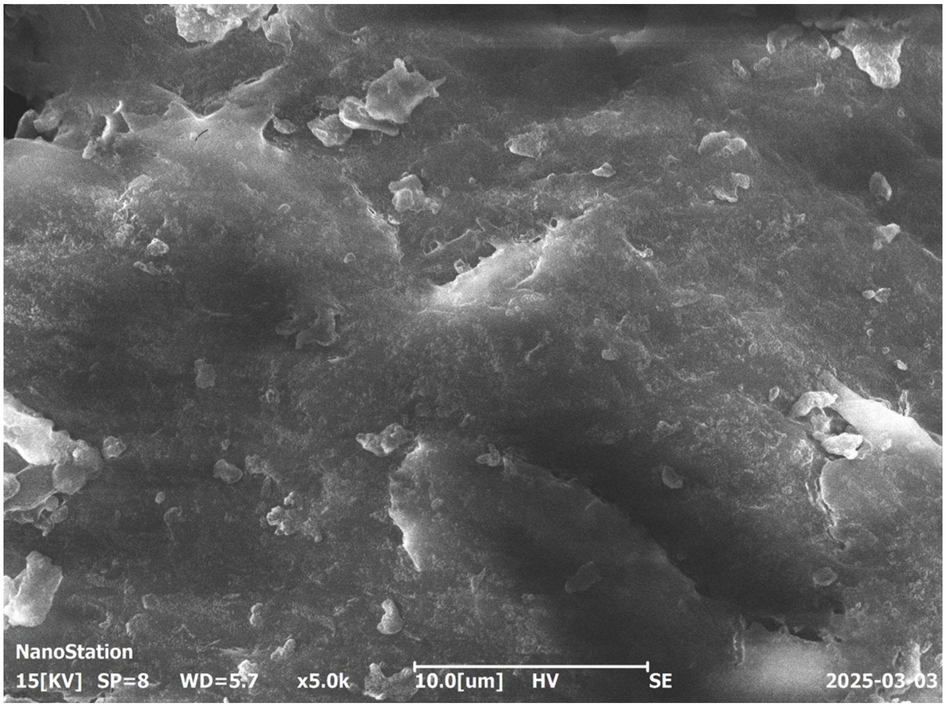

(a) Cross sectional SEM image of pure hydrogel

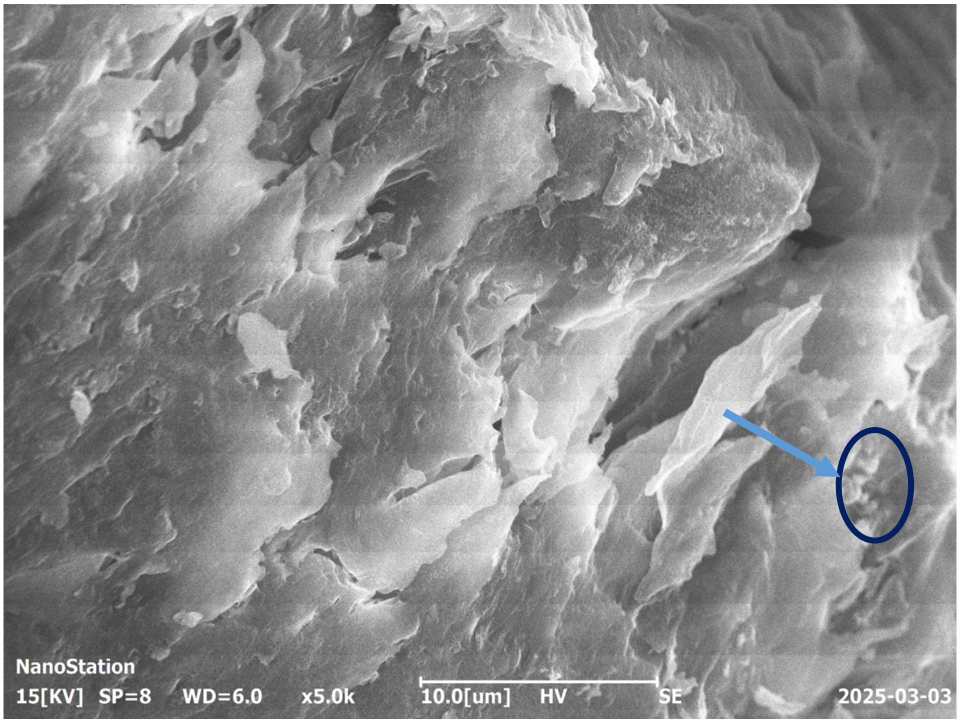

(b) Cross sectional SEM image of ZnO-doped hydrogel

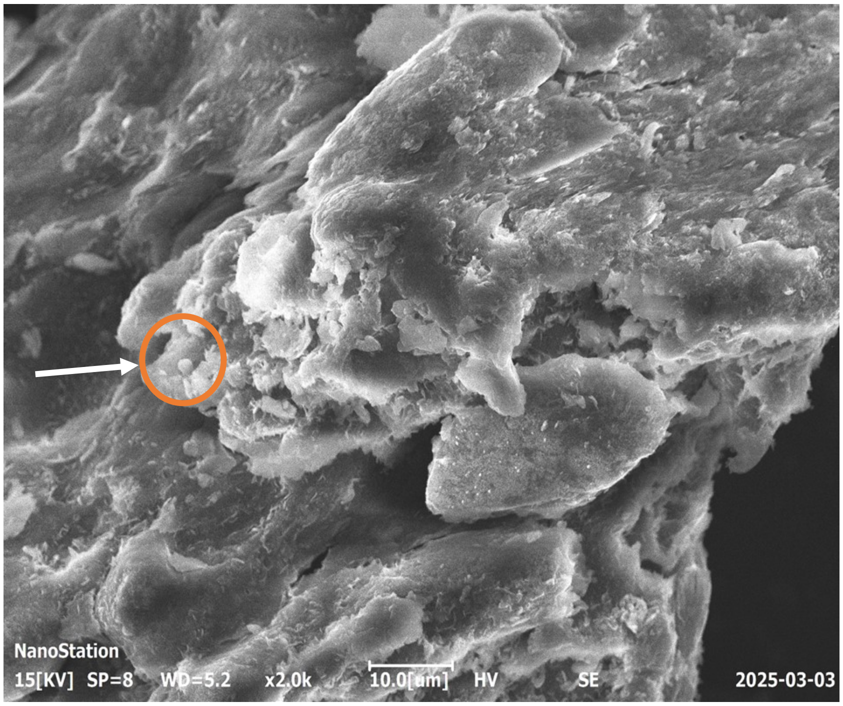

(c) Cross sectional SEM image of CuO-doped hydrogel

S4 UV graphs along raw data for pH study, photolysis, adsorption study of ZnO-doped hydrogel and CuO-doped hydrogel.

CuO at Iltaf pH3 last.sca   CuO at Iltaf pH7.sca   CuO at Iltaf pH9.sca

ZnO at Iltaf pH9 last.sca   ZnO at Iltaf pH3 last.sca   ZnO at Iltaf pH7 last.sca

photolysis.sca   pure mb 5 pm.sca   adsorption cu.sca   adsorption zn.sca

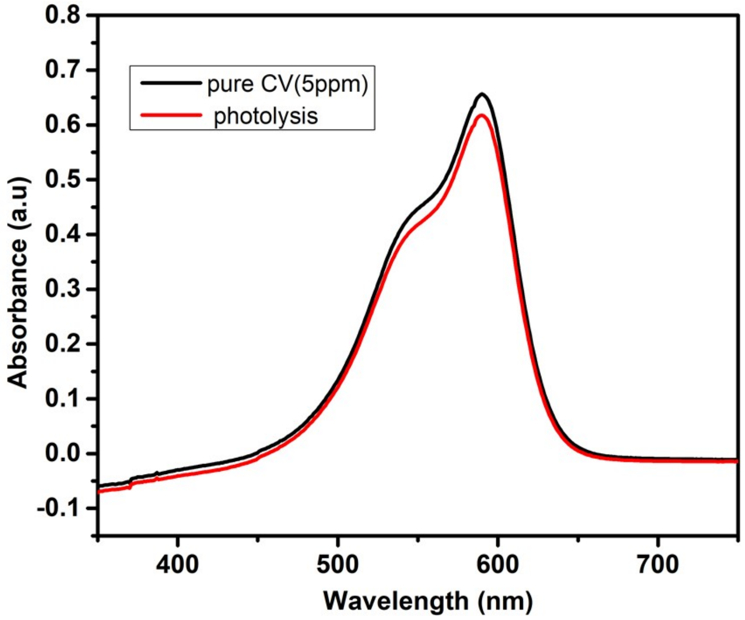

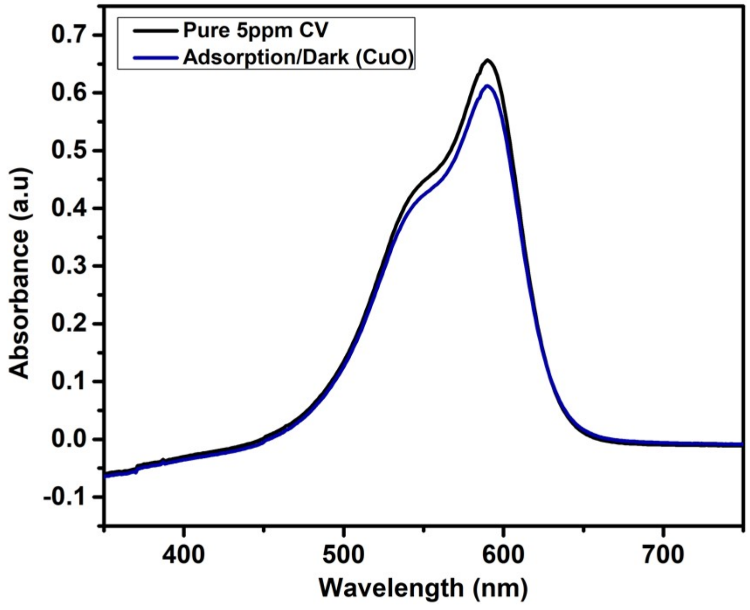

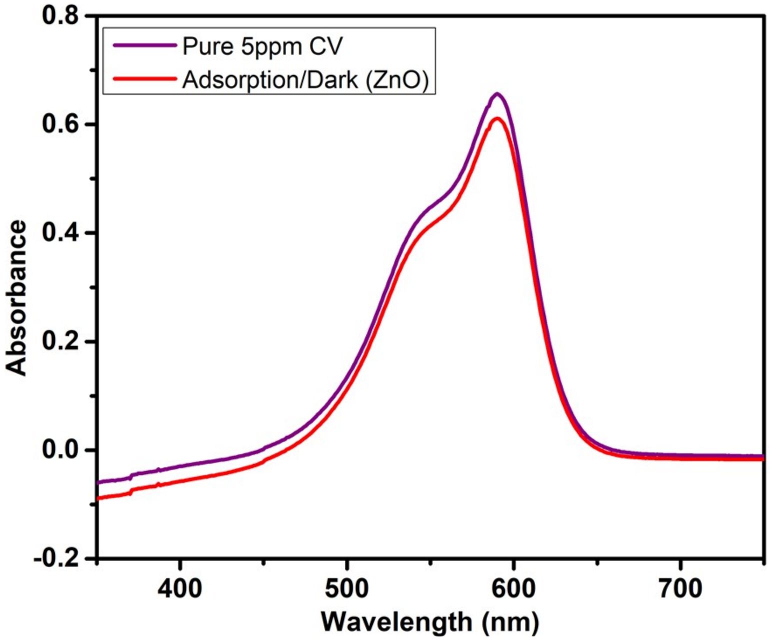

S5 XRD raw data for ZnO-doped (HPBA-Z) hydrogel and CuO-doped (HPBA-C) hydrogel.

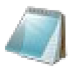

HPBA-C#3##20250409-111814_100.mdi

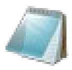

HPBA-Z#4##20250409-115027_100.mdi

S6. FTIR raw data for barren (PBA) (no photocatalyst), and ZnO-doped (HPBA-Z) hydrogel and CuO-doped (HPBA-C) hydrogel.

PBA.spa HPBA Z.spa HPBA C (1).spa

S7. To show the reliability and reproducibility of the results.

In this study, each photocatalytic experiment was performed once under firmly same and optimized settings due to instrumental and sample limitations. However, the experimental procedure was carefully standardized, and initial optimization trials were performed to confirm reproducibility and reliability of the results. Multiple absorbance readings were noted at each irradiation interval to minimize instrumental error and confirm stability of the measurements. Therefore, while standard deviation values could not be statistically calculated, the observed trends and performance comparisons between CuO- and ZnO-doped hydrogel nanocomposites remain reliable and representative. Future work will include triplicate measurements and statistical error analysis to further authenticate the photocatalytic data.

S8 BET raw data for CuO and ZnO-doped hydrogel composites

4.PDF 5.PDF

CuO ZnO
